# Statin-Induced Geranylgeranyl Pyrophosphate Depletion Promotes PCSK9–Dependent Adipose Insulin Resistance

**DOI:** 10.3390/nu14245314

**Published:** 2022-12-14

**Authors:** Xin Shu, Jiaqi Wu, Tao Zhang, Xiaoyu Ma, Zuoqin Du, Jin Xu, Jingcan You, Liqun Wang, Ni Chen, Mao Luo, Jianbo Wu

**Affiliations:** 1 Drug Discovery Research Center, Southwest Medical University, Luzhou 646000, China; 2 The People’s Hospital of Leshan, Leshan 614000, China; 3 Laboratory for Cardiovascular Pharmacology, Department of Pharmacology, School of Pharmacy, Southwest Medical University, Luzhou 646000, China; 4 Metabolic Vascular Disease Key Laboratory of Sichuan Province, Southwest Medical University, Luzhou 646000, China; 5 Luzhou Municipal Key Laboratory of Thrombosis and Vascular Biology, Southwest Medical University, Luzhou 646000, China

**Keywords:** obesity, statin, PCSK9, insulin resistance, adipose tissue

## Abstract

Statin treatment is accepted to prevent adverse cardiovascular events. However, statin therapy has been reported to be dose-dependently associated with increased risk for new-onset type 2 diabetes mellitus (T2DM). Proprotein convertase subtilisin/kexin type 9 (PCSK9) is expressed in adipose tissue and is positively correlated with lipid metabolism. It is, however, unknown if PCSK9 participates in adipocyte insulin resistance occurring as a result of statin use. Our goal was to use an in vitro adipose tissue explant approach to support the hypothesis that PCSK9 regulates statin-induced new-onset T2DM. Studies were performed using Pcsk^−/−^ and C57Bl/6J control mice. Pcsk9^−/−^ and control mice were fed a high-fat diet to affect a state of chronically altered lipid metabolism and increased PCSK9. Epididymal fat was excised and incubated with atorvastatin (1 µmol/L) in the absence and presence of insulin or geranylgeranyl pyrophosphate (GGPP). PCSK9 mRNA was evaluated using quantitative rtPCR. We further examined the effects of atorvastatin on insulin-mediated AKT signaling in adipose tissue explants by immunoblotting. Atorvastatin was found to upregulate PCSK9 gene expression in adipose tissue. The metabolic intermediate GGPP is required to downregulate PCSK9 expression. PCSK9 deficiency protects against statin-induced impairments in insulin signaling. Moreover, supplementation with GGPP reversed atorvastatin-induced suppression of insulin signaling. Furthermore, the basal and atorvastatin-stimulated release of free fatty acids was observed in adipose tissue from wild-type mice but not PCSK9 deficient mice. Collectively, we describe a novel mechanism for PCSK9 expression in adipose tissue that could mediate statin-impaired adipose insulin resistance.

## 1. Introduction

Statins inhibit 3-hydroxy-3-methylglutaryl-CoA reductase (HMGCR), reduce cholesterol biosynthesis, and increase LDL receptor (LDLR) expression [1]. Although statin therapy appears to reduce primary and secondary cardiovascular events, multiple meta-analyses of clinical trials have reported that statin therapy is dose-dependently associated with a subsequent increased risk of new-onset type 2 diabetes mellitus (T2DM) [2,3,4,5]. The incidence of new-onset T2DM is also related to genetic HMGCR variants, indicating a role for gene-specific associations with cholesterol metabolic risk of T2DM development [6]. Statins impair insulin signaling causing statin-induced insulin resistance in adipose tissue, coincident with activation of NLR family pyrin domain-containing 3 (NLRP3) inflammasome and impaired insulin-stimulated lipogenesis in adipocytes [7,8]. In addition, statins catalyze the synthesis of mevalonate that generates geranylgeranyl diphosphate (GGPP). Statin-mediated reductions in GGPP levels are partly responsible for decreased insulin signaling [7]. Thus, statin treatment correlates the mechanism underlying new-onset T2DM induction with GGPP depletion.

Proprotein convertase subtilisin/kexin type 9 (PCSK9) is a plasma protein that binds to the low-density lipoprotein receptor (LDLR) and preferentially targets it for endosomal degradation. Inhibition of PCSK9 has emerged as an effective approach for regulating LDL-C levels and is, thus, consistent with reducing CVD events [9,10]. Despite substantial discoveries relating to PCSK9 genetic variants being associated with an increased risk of T2DM [3], several experimental studies and clinical trials have reported conflicting results regarding plasma PCSK9 levels and T2DM [11,12,13]. PCSK9 deficiency is associated with impaired glucose tolerance in mice, which appears to be the consequence of decreased insulin secretion from the pancreas rather than insulin resistance [14]. Overall, the impact of PCSK9 on glucose metabolism remains unclear.

Statins can influence the regulation of PCSK9 through a complex regulatory process [15]. By inhibiting cholesterol biosynthesis, statins increase the nuclear translocation of sterol regulatory element-binding protein-2 (SREBP2), upregulate the cellular LDLR and PCSK9 genes, and lead to an elevation of circulating PCSK9 levels [16,17]. Statins also stimulate gene and protein expression of hepatocyte nuclear factor 1α (HNF1α), a further transcriptional activator of PCSK9, which results in a higher induction of PCSK9 compared with that of LDLR [18]. It is unknown whether PCSK9 affects adipose tissue insulin resistance as a result of statin treatment.

Besides the liver, PCSK9 has been shown to be expressed in adipose tissues and is positively correlated with the body mass index [19]. PCSK9 plays a critical role in lipid metabolism and downregulates triglycerides and free fatty acids (FFAs) into visceral adipocytes, likely through adipose tissue VLDLR [5]. We, therefore, compared the response of adipose tissue explants from wild-type (WT) and PCSK deficient (Pcsk9^−/−^) mice to statin (artorvostatin) treatment. Control mice were also fed a high-fat diet to affect a state of chronically altered lipid metabolism and increased PCSK9. Ex vivo analyses demonstrated statin-induced upregulation of PCSK9 gene expression in adipose tissues and attenuated insulin-stimulated AKT signaling in the adipose tissue of WT mice but not Pcsk9^−/−^ mice. Furthermore, geranylgeranyl isoprenoids (GGPP) were shown to be required to impair insulin signaling in the adipose tissue of WT mice. Consistent with these results, an increase in free fatty acid production was observed in the adipose tissue of Pcsk9^−/−^ mice.

## 2. Materials and Methods

### 2.1. Reagents

Atorvastatin was purchased from Gödecke/Parke-Davis (Freiburg, Germany). GGPP was obtained from GlpBio (Montclair, NJ, USA). GGPP ELISA kit was from Shanghai Win-win Biotechnology (Shanghai, China). The FFA assay kit was purchased from Abcam (Cambridge, MA, USA).

### 2.2. Animals

C57BL/6J mice were obtained from Chongqing Tengxin Bioscience Inc. (Chongqing, China). Pcsk9^−/−^ mice (strain: B6.129S6-PCSK9^tm1jdh^/J; Stock number: 005993) were purchased from the Jackson Laboratory (Bar Harbor, ME, USA). All protocols for animal use were reviewed and approved by the Animal Care Committee of Southwest Medical University in accordance with Institutional Animal Care and Use Committee guidelines (Project identification code: 2020YJ0340).

### 2.3. HFD-Fed Mouse Model

Eight-week-old male Pcsk9^−/−^ and C57BL/6J mice were fed a high-fat diet (HFD) (TP2330055A; fat calories 60%, carbohydrate calories 25%, and protein calories 15%; Trophic Animal Feed High-tech Co. Ltd., Nantong, China) for 16 weeks. Age-matched male mice that were fed a normal diet (TP2330055AC; fat calories 10%, carbohydrate calories 75%, and protein calories 15%; Trophic Animal Feed High-tech Co. Ltd., Nantong, China) were used as controls. Blood samples were obtained from the tail vein and blood glucose levels were measured using an automatic glucometer (Accu-Chek; Roche Diagnostics, Mannheim, Germany).

### 2.4. Experimental Design

Mice were killed by cervical dislocation. As described previously [8], epididymal adipose tissues (EAT) were isolated and minced into ~5 mg pieces in DMEM containing 10% FBS. After 2 h of incubation, 50 mg of small pieces were placed in serum-free DMEM and exposed to 1 µmol/L atorvastatin for 18 h, and then stimulated with 100 nM insulin (Sigma-Aldrich, St. Louis, MO) for 10 min. In specific experiments, EAT explants were also treated with insulin (100 nM, 4 h) or GGPP (5μM, 50μM; GlpBio); added to the culture medium at the same time as atorvastatin. Adipose tissue lysates were subsequently used for measurements related to insulin signaling by immunoblotting, GGPP by ELISA and free fatty acids (FFAs).

### 2.5. Quantitative PCR (qPCR)

EAT was collected, and qPCR analysis was performed as previously described [20]. The total RNA was extracted from frozen samples using TRIzol reagent (Invitrogen, Carlsbad, CA, USA). RNA samples were pre-treated with deoxyribonuclease I (Invitrogen Life Technologies, Carlsbad, CA, USA), and a SuperScript kit (Invitrogen Life Technologies, Carlsbad, CA, USA) was used to synthesize cDNA according to the manufacturer’s recommendations. qPCR was performed using miScript SYBR Green PCR Kits (Qiagen). Amplification was detected on an ABI PRISM 7700 cycler (Applied Biosystems, Foster City, CA). Each sample was measured in duplicate, and the results were normalized to GAPDH. The oligonucleotide sequences of the PCR primers were: (1) mouse PCSK9 (CAGGGAGCACATTGCATCC, TGCAAAATCAAGGAGCATGGG); (2) mouse GAPDH (TTCACCACCATGGAGAAGG, CTCGTGGTTCACACCCATC). The relative fold changes in target gene expression were calculated using the 2−ΔΔCT method.

### 2.6. Immunoblotting

EAT lysates were prepared, and equal amounts of protein were subjected to SDS-PAGE and transferred to polyvinylidene difluoride membranes by electroblotting. After blocking, the membranes were incubated with antibodies directed against phospho-Akt (Ser473, #4060), and total AKT (#4691) (Cell Signaling Technology, Massachusetts, USA). The secondary antibody was horseradish-peroxidase (HRP)-conjugated goat IgG raised against IgG (#58802; Cell Signaling Technology, Massachusetts, USA). Blots were developed with enhanced chemiluminescence (ECL) substrate (Pierce). The band intensity was measured by using IMAGE J software.

### 2.7. ELISA

EATs were obtained and homogenized in a lysis buffer. The supernatants were extracted to measure GGPP concentration by using an ELISA kit (Shanghai Win-win Biotechnology Co., Ltd., Shanghai, China) according to the manufacturer’s instructions. GGPP values were measured at a wavelength of 450 nm on a microplate reader. FFAs levels were measured by a specific enzymatic assay kit (Abcam, ab65341; Shanghai, China). The values were calculated by performing on a microplate reader at OD 570 nm. Experimental values were compared with standard values. All values were normalized to total cellular protein, which was determined using a BCA assay, and expressed as ng/mg protein.

### 2.8. Statistical Analysis

Data are presented as mean ± standard error of the mean. Statistical differences among groups were analyzed by one-way analysis of variance with a post hoc test applied to identify individual comparisons. *p* value less than/equal to 0.05 was set as statistical significance.

## 3. Results

### 3.1. Atorvastatin Upregulates PCSK9 mRNA in Adipose Tissue

**Figure 1:**
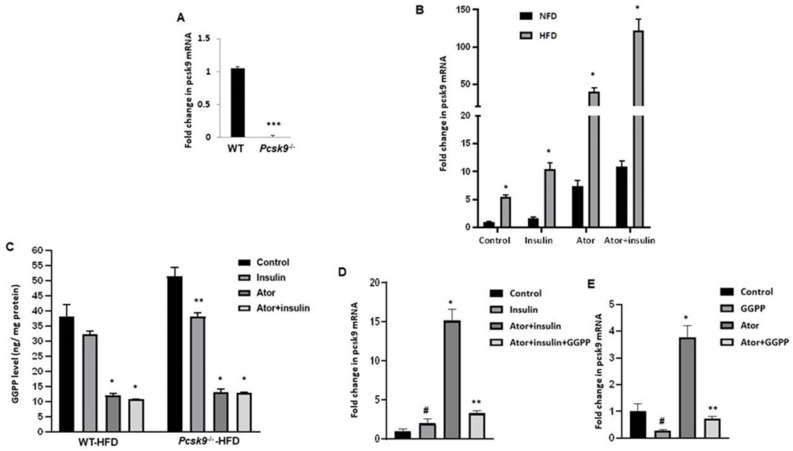
Atorvastatin upregulates PCSK9 mRNA in adipose tissue. (**A**) Quantitative RT-PCR analysis of PCSK9 mRNA in EAT from WT and Pcsk9^−/−^ mice. n = 3 mice per group. *** *p* < 0.001 Vs. WT mice. Data are mean ± SEM. (**B**) Quantitative RT-PCR analysis of total RNA isolated from EAT for PCSK9 mRNAs. Data were normalized to the amount of GAPDH mRNA and expressed relative to the corresponding WT-NFD. n = 6 mice per group. * *p* < 0.05 Vs. WT-NFD mice. Data are mean ± SEM. (**C**) GGPP levels were measured by ELISA (n = 6 per group). * *p* < 0.05 Vs. Control and insulin group in WT-HFD mice; ** *p* < 0.01 Vs. Control in Pcsk9^−/−^-HFD mice. Data are mean ± SEM. (**D**) Quantitative RT-PCR analysis of total RNA isolated from EAT for PCSK9 mRNAs. * *p* < 0.05 Vs. Control and insulin group; ** *p* < 0.01 Vs. Ator + insulin; ^#^ *p* < 0.05 Vs. Control. Data were normalized by the amount of GAPDH mRNA and expressed relative to the corresponding control. n = 6 per group. Data are mean ± SEM. (**E**) Quantitative RT-PCR analysis of PCSK9 mRNAs. * *p* < 0.05 Vs. Control and insulin group; ** *p* < 0.01 Vs. Ator; ^#^ *p* < 0.05 Vs. Control. Data were normalized by the amount of GAPDH mRNA and expressed relative to the corresponding control. n = 6 micper group. Data are mean ± SEM. Ator, atorvastatin.

We first studied the effect of statin treatment on PCSK9 mRNA. qPCR analysis of EAT confirmed the expression of PCSK9 mRNA from WT mice (Figure 1A). Small pieces of EAT were incubated (18 hrs) with 1 μmol/L atorvastatin. As shown in Figure 1B, PCSK9 mRNA expression was increased by approximately 5-fold in adipose tissue from HFD compared with that in control diet (NFD) fed mice. In addition, PCSK9 mRNA expression was enhanced by insulin (100 nM) and was significantly higher in adipose tissue from HFD compared with NFD mice. Amongst the various treatments, the highest level of PCSK9 mRNA expression was observed following exposure to the combination of atorvastatin and insulin.

Statins block HMG-CoA reductase, which catalyzes the synthesis of mevalonate that generates GGPP. To assess whether PCSK9 expression upregulates the mevalonate pathway, we further examined GGPP levels in adipose tissue from HFD mice by ELISA. As shown in Figure 1C, in obese mice, both WT-HFD and PCSK9^−/−^-HFD showed high basal levels of GGPP in EAT. No apparent statistical difference was evident between the two groups of mice given the HFD. However, GGPP levels were significantly decreased following treatment with atorvastatin alone or in combination with insulin.

To evaluate whether atorvastatin-induced PCSK9 mRNA is due to GGPP depletion, we performed additional experiments adding back GGPP. The results show that the addition of GGPP completely abrogates the effects of atorvastatin or atorvastatin + insulin on the induction of PCSK9 mRNA (Figure 1D,E). GGPP alone had an anti-inhibitory effect on the expression of PCSK9 mRNA (Figure 1E). These findings are consistent with the mechanism of action of the statin action being mediated through GGPP depletion.

### 3.2. PCSK9 Deficiency Protects Statin-Inhibited Insulin Signaling in Adipose Tissue

**Figure 2:**
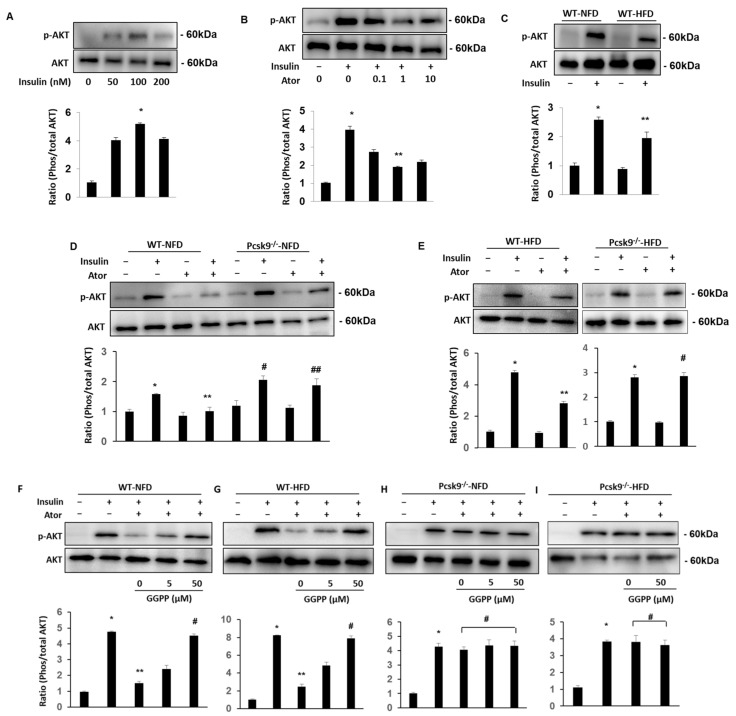
PCSK9 deficiency protects statin-inhibited insulin signaling in adipose tissue. (**A**) AT explants were treated with insulin (0, 50, 100, and 200 nM) for 10 min, then the lysates were detected with Western blotting. Representative images (top) and quantification (bottom) of EAT from WT-HFD mice as indicated. * *p* < 0.05 Vs. Control. (**B**) Pretreatment of atorvastatin (0, 0.1, 1, and 10 μmol/L) for 18 h, and then exposed to insulin (100 nM) for 10 min. Representative images (top) and quantification (bottom) of EAT from WT-HFD mice as indicated. * *p* < 0.05 Vs. Control; ** *p* < 0.01 Vs. Ator at 0, and 0.1μmol/L. (**C**) AT explants were treated with insulin (100 nM) for 10 min. Representative images (top) and quantification (bottom) of EAT from WT-NFD and WT-HFD mice as indicated. * *p* < 0.05 Vs. Control; ** *p* < 0.01 Vs. insulin in WT-NFD. (**D**)The treatment of insulin (100 nM) for 10 min stimulated phosphorylation of AKT after pretreatment with atorvastatin (1 μmol/L) for 18 h in EAT explants from WT and Pcsk9^−/−^ mice. Representative images of pAKT/AKT (top) and quantification (bottom) of EAT from NFD as indicated. All graphs correspond to the blots above them and represent densitometric analyses of 3 independent experiments. * *p* < 0.05 Vs. Control; ** *p* < 0.01 Vs. insulin; ^#^ *p* < 0.05 Vs. insulin; ^##^ *p* < 0.05 Vs. Ator. (**E**) Representative immunoblots and quantification of EAT from HFD as indicated. All graphs correspond to the blots above them and represent densitometric analyses of 3 independent experiments. * *p* < 0.05 Vs. Control; ** *p* < 0.01 Vs. Control, insulin, and Ator; ^#^ *p* < 0.05 Vs. Ator. (**F**–**I**) Representative images and quantification of insulin-mediated pAKT after treatment of EAT explants with atorvastatin plus supplementation with and without GGPP at the dose indicated. NFD (**F**) and HFD-fed (**G**) groups in WT mice as shown, * *p* < 0.05 Vs. Control; ** *p* < 0.01 Vs. insulin; ^#^ *p* < 0.05 Vs. insulin + Ator. NFD (**H**) and HFD-fed (**I**) groups in Pcsk9^−/−^ mice as shown, * *p* < 0.05 Vs. Control; ^#^ *p* < 0.05 Vs. insulin. n = 3 mice/per group. All graphs correspond to the blots above them and represent densitometric analyses of 3 independent experiments. Data are mean ± SEM. Ator, atorvastatin.

To define the role of PCSK9 in obesity in vivo, we fed mice high-fat chow for 16 weeks, which produced obesity and hyperglycemia (Figure S1). To examine whether PCSK9 plays a role in the regulation of insulin sensitivity in EAT, AT explants were treated with insulin (0, 50, 100, and 200 nM) for 10 min. Biochemical events related to insulin signaling were then examined in tissue lysates using Western blotting. The results show that AKT activity (ratio of p-AKT to total AKT) in EAT explants was highest in cells treated with 100 nM insulin (Figure 2A). Next, we used atorvastatin (0, 0.1, 1, and 10 μmol/L) to evaluate the effects of AKT signaling after insulin treatment. We chose to use insulin at a concentration of 100 nM since this concentration had resulted in the largest change in the p-AKT to AKT ratio. The results showed that after pretreatment with 1 or 10 μmol/L atorvastatin for 18 hours, the ratio of p-AKT to total AKT was significantly decreased compared with those in the 0.1  μmol/L atorvastatin or control group (Figure 2B). We further found that insulin-mediated AKT phosphorylation was significantly decreased in HFD WT EAT explants compared with that in NFD WT mice (Figure 2C).

Treatment with insulin (100 nM; 10 min) stimulated phosphorylation of AKT in EAT explants from WT and Pcsk9^−/−^ mice. The levels of AKT phosphorylation were markedly higher in tissue (Figure 2D) from Pcsk9^−/−^-ND mice compared with those of WT-NFD mice. Pretreatment with atorvastatin (18 hrs) significantly inhibited insulin-stimulated AKT phosphorylation in EAT from NFD (Figure 2D) and HFD-fed control mice (Figure 2E), but not in Pcsk9^−/−^ mice.

To further support the inhibitory effects of atorvastatin being due to GGPP depletion, we performed experiments where GGPP was added back. Supplementation with GGPP at 50 μM (but not 5 μM) completely restored atorvastatin-induced suppression of insulin-stimulated phosphorylation of AKT in NFD (Figure 2F) and HFD-fed (Figure 2G) control mice but not in Pcsk9^−/−^ mice (Figure 2H,I). These results suggest that PCSK9 deficiency would be associated with improved insulin sensitivity in adipose tissue, and GGPP could completely restore statin-induced impairments in insulin signaling.

### 3.3. PCSK9 Deficiency Decreases Statin-Stimulated FFAs in Adipose Tissue

**Figure 3:**
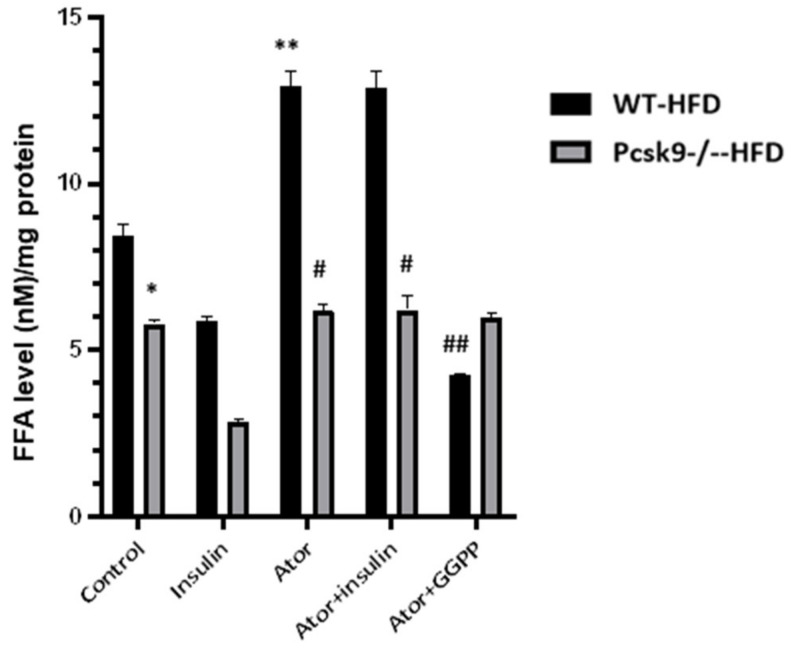
PCSK9 deficiency decreases statin-stimulated FFAs in adipose tissue. The FFAs levels of EAT were measured by ELISA. n = 6 mcie per group. * *p* < 0.05 Vs. Control in WT-HFD mice. ** *p* < 0.05 Vs. Control in WT-NFD mice; ^#^ *p* < 0.05 Vs. Ator or insulin + Ator in WT-HFD mice; ^##^ *p* < 0.05 Vs. Ator in WT-HFD mice. Data are mean ± SEM.

Adipose tissue insulin resistance is associated with the excess release of FFAs through lipolysis. We, therefore, evaluated the effect of statins on the intracellular accumulation of FFAs in adipose tissue. Following feeding with the HFD, PCSK9 deficiency is correlated with decreased basal FFAs production compared with that of control mice. Accumulation of FFAs in EAT from the HFD diet-fed mice was significantly increased by the exposure to atorvastatin (Figure 3). This increase in FFAs content was blocked by supplementation with GGPP, suggesting that GGPP is related to statin-mediated lipolysis. We also found that insulin treatment reduced FFAs production compared with control in WT mice. Furthermore, insulin-stimulated production of FFAs in Pcsk9^−/−^ mice was significantly less than that observed in WT mice, suggesting that PCSK9 is linked to statin-mediated lipolysis.

## 4. Discussion

In this study, we found that PCSK9 plays a crucial role in statin-induced insulin resistance in adipose tissue. The supplementation of GGPP diminishes basal and statin-induced upregulation of pcsk9 expression. PCSK9 deficiency prevented statin-induced adipose insulin resistance. Furthermore, supplementation with GGPP restored atorvastatin-induced suppression of insulin-AKT signaling in adipose tissue. Collectively, our data suggest a novel mechanism for PCSK9 expression in adipose tissue that could underlie statin-impaired insulin adipose resistance.

Our data provide genetic evidence, in tissue explants, that atorvastatin impairs insulin-mediated signaling in adipose tissue via PCSK9. Specifically, we found that phosphorylation of AKT through phosphorylation (Ser473) was decreased when adipose tissue was exposed to atorvastatin plus insulin compared with that in the presence of insulin alone. This effect was evident in adipose tissue from WT mice fed either a normal or HFD. In agreement with this relationship, we found that PCSK9 mRNA is also upregulated by insulin in adipose tissue. The study by Lalli et al. did not show that statins decreased insulin resistance in HFD-fed animals, but rather that statins improved insulin signaling by mediating the IRS-1/PI3K/Akt pathway [21]. However, our data did not support this mechanism because we observed that statin treatment leads to reduced insulin-AKT signaling in adipose tissues from ex vivo, which contradicts the previously mentioned observation in the liver and muscle. The discrepancy in outcome from statin-mediated insulin signaling was likely related to tissue-specific dependence. Future studies are necessary to define the relationship more precisely between adipose tissue and whole-body insulin resistance in the treatment of statins from diet-induced obesity.

Previous studies have shown that GGPP is an endogenous regulator of adipocyte function [22]. GGPP is essential in maintaining adipocyte survival by regulating apoptosis (23). Moreover, adipose-specific mevalonate pathway-disrupted (aKO) mice exhibit severe dysfunction of glucose metabolism [23]. GGPP was also found in the liver, and liver-specific GGPP deletion improved systemic glucose tolerance but not insulin sensitivity [24]. Insulin signaling in adipocytes suppresses AT lipolysis, decreasing the flux of FFAs to the blood [20]. Consistent with this, we found that the treatment with atorvastatin in HFD mice significantly increased the accumulation of FFAs. This increase in FFA content was reversed by supplementation of GGPP, suggesting that GGPP is linked to statin-mediated lipolysis.

Atorvastatin attenuates adipocyte maturation and glucose transporter expression by inhibiting isoprenoid biosynthesis and impairs glucose tolerance [25,26]. Genetic PCSK9 deficiency has been shown to lead to adipocyte hypertrophy in white adipose tissue, independently of LDLR, and in the presence of a higher fatty acid uptake [18]. In patients with cardiovascular disease, PCSK9 expression was positively correlated with epicardial adipose tissue thickness and inflammation [27,28], associated with the direct action of statins on the EAT secretory profile [29], suggesting that EAT inflammation could be associated with the local PCSK9 levels, regardless of circulating PCSK9 levels. Supplementation with GGPP can reverse the increased atorvastatin-induced expression of PCSK9. We further suggest that GGPP-dependent AKT signaling is required for insulin stimulation in the WT type. The current finding is consistent with a recent observation that a statin-mediated reduction in a geranylgeranyl isoprenoid is a requirement for impaired insulin signaling [13]. Our results thus suggest that the statin-induced PCSK9 expression could be attributed to GGPP depletion (i.e., statin-mediated) in adipose tissues. An increased level of PCSK9 from adipose tissue was shown in HFD mice compared with NFD mice. The GGPP effect was identical in the presence or absence of statin in these conditions, indicating that GGPP acts directly on the level of PCSK9 mRNA in adipose tissues. Additional experiments will be necessary to clarify the potential importance of future studies.

STUDY LIMITATIONS. While our study provides a potential cause-and-effect relationship between PCSK9 and statin-induced suppression of insulin signaling, there are limitations in the ability of any murine study to exactly reproduce human pathology. Furthermore, our study has yet to identify the molecular and cellular pathways by which statins regulate PCSK9 for systemic insulin resistance in vivo, though we have shown the important and intriguing observation that GGPP is required for restoring atorvastatin-induced suppression of insulin-AKT signaling in adipose tissue. Whether reducing local adipose tissue PCSK9 levels or small RHo-GTPase has an essential effect on statin-induced new-onset diabetes needs further investigation. Additional studies are warranted to resolve these critical issues. Furthermore, our study was limited to adipose tissue from male mice, and, therefore, future studies should consider whether the observations are also directly applicable to females.

In summary, our experiments highlight a previously uncharacterized role of atorvastatin in insulin-mediated adipose tissue resistance. Specifically, we have shown that atorvastatin upregulates the expression of PCSK9 mRNA, and GGPP restored atorvastatin-induced suppression of insulin-AKT signaling in adipose tissue.

## Data Availability

The datasets used and/or analyzed during the current study are available from the corresponding author on reasonable request.

## Supplementary Materials

The following supporting information can be downloaded at: https://www.mdpi.com/article/10.3390/nu14245314/s1, Figure S1: High-fat diet induces obesity, and hyperglycemia in C57BL/6J mice.
