# Supplementary material for: Statin-Induced Geranylgeranyl Pyrophosphate Depletion Promotes PCSK9–Dependent Adipose Insulin Resistance
Source: Nutrients. 2022 Dec 14;14(24):5314. doi: 10.3390/nu14245314 (PMC9853319; doi:10.3390/nu14245314)

Supplementary Materials

Figure S1. High-fat diet induces obesity, and hyperglycemia in C57BL/6J mice. Mice were fed high-fat diet (HFD) or normal diet (NFD) for 16 weeks, after which (A) body weight, and (B) plasma glucose were measured (n=6/group); *P<0.05 vs. NFD group.

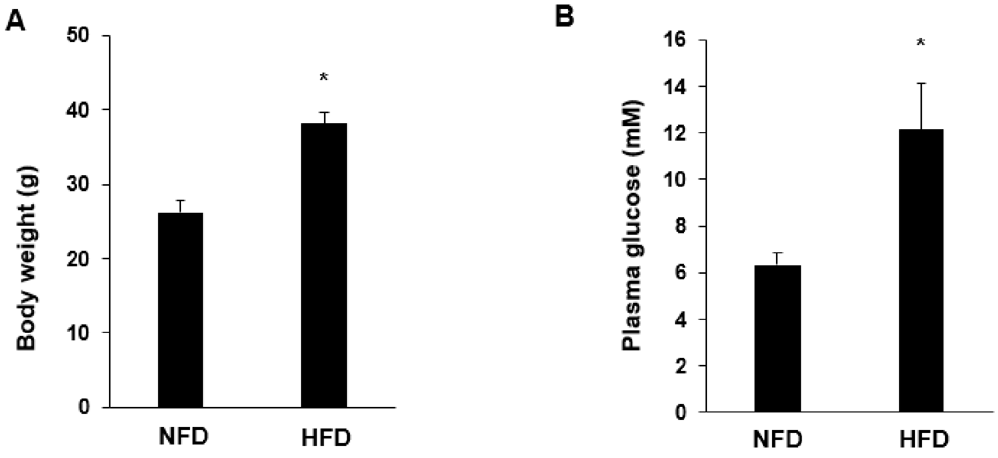

Supplement: Supplementary file 1 [file nutrients-14-05314-s001.zip › nutrients-2030863-supplementary.pdf]
